# Elevated CO_2_ modulates airway contractility

**DOI:** 10.1098/rsfs.2020.0021

**Published:** 2021-02-12

**Authors:** Masahiko Shigemura, Jacob I. Sznajder

**Affiliations:** Division of Pulmonary and Critical Care Medicine, Northwestern University, Chicago, IL, USA

**Keywords:** carbon dioxide (CO_2_), hypercapnia, respiratory acidosis, lung airways, airway smooth muscle contractility

## Abstract

Carbon dioxide (CO_2_), a primary product of oxidative metabolism, can be sensed by eukaryotic cells eliciting unique responses via specific signalling pathways. Severe lung diseases such as chronic obstructive pulmonary disease are associated with hypoventilation that can lead to the elevation of CO_2_ levels in lung tissues and the bloodstream (hypercapnia). However, the pathophysiological effects of hypercapnia on the lungs and specific lung cells are incompletely understood. We have recently reported using combined unbiased molecular approaches with studies in mice and cell culture systems on the mechanisms by which hypercapnia alters airway smooth muscle contractility. In this review, we provide a pathophysiological and mechanistic perspective on the effects of hypercapnia on the lung airways and discuss the recent understanding of high CO_2_ modulation of the airway contractility.

## 1. Introduction

Cells and tissues sense and respond to changes in the concentration of gaseous molecules through specific signalling pathways. Oxygen- and nitric oxide-activated cellular signalling pathways have been extensively studied [1–3], but much less is known about the mechanisms by which non-excitable cells sense and respond to changes in carbon dioxide (CO_2_) concentrations [3–5]. CO_2_ is a primary product of oxidative metabolism and its physiological levels in mammals are significantly higher than atmospheric levels (approx. 5% versus approx. 0.04%, https://scripps.ucsd.edu/programs/keelingcurve/) [4,6], suggesting that CO_2_ is inextricably linked to physiological conditions. In humans, the elevation of CO_2_ levels in tissues and the bloodstream (hypercapnia) is a consequence of inadequate alveolar gas exchange in patients with lung diseases such as the acute respiratory distress syndrome (ARDS) [7–9], chronic obstructive pulmonary disease (COPD) [10–12] and others [13–15]. In clinical situations, hypercapnia has been initially proposed to be benign or even protective in the lung since hypercapnia and its associated acidosis have been shown to attenuate systemic cytokine response in mechanically ventilated patients with acute lung injury and ARDS [7,8,16]. However, it is becoming increasingly evident that elevated CO_2_ conditions have deleterious pathophysiological effects on various organs, including the lung [9,17–19], skeletal muscles [20–22] as well as innate immunity system [18,23–27]. In the lung, recent studies suggest that high concentrations of CO_2_ activate specific gene expression [19,28,29] and signal transduction pathways with adverse consequences on alveolar epithelial function (alveolar fluid clearance) [17,30–35] and epithelial cell repair [36–39].

Hypercapnia is also reported to modulate the tone of lung airways which is a dynamic equilibrium between various excitatory and inhibitory mechanisms. The effects of hypercapnia on the airways and airway smooth muscle are controversial, as there are reports attesting to it causing increased airway contractility [19,40–49] or airway relaxation [50–61]. Here, we review recent advances in our understanding of how elevated CO_2_ conditions modulate the airway tone, focusing on the effects of hypercapnia and respiratory acidosis.

## 2. Hypercapnia-induced bronchoconstriction

**Figure 1.**
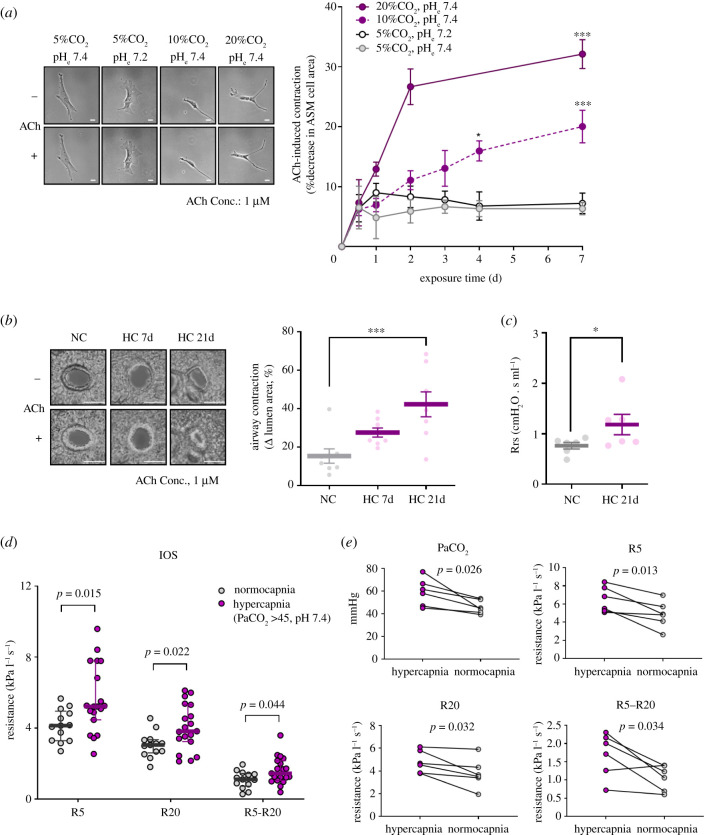
Normoxic hypercapnia increases airway smooth muscle contractility. (*a*) Acetylcholine (ACh)-induced cell contraction in mouse airway smooth muscle cells exposed to different conditions. Left, representative images from 7-day exposure conditions (scale bar, 50 µm). Right, time-course quantification of ACh-induced cell contraction. (*b,c*) C57BL/6 J wild-type mice were exposed to 21% O_2_ and 10% CO_2_ (HC) or maintained in room air (NC) for up to 21 days. Representative images (top; scale bar, 100 µm) and quantification (bottom) of ACh-induced airway contraction in precision-cut lung slices (*b*). Total resistance of the respiratory system (Rrs) at baseline on a FlexiVent instrument (*c*). (*d*) Comparison of respiratory resistance measured by impulse oscillometry between normocapnic and hypercapnic patients with chronic stable COPD. Values of R5, R20 and R5–R20 indicate total, proximal and peripheral respiratory resistance, respectively. (*e*) Changes of respiratory resistance in hypercapnic patients. All data are expressed as means ± s.e.m. (*a*–*c*) or median with interquartile range (*d,e*). *p < 0.05, ***p < 0.001. Reproduced from [19]. Copyright © 2018 American Association for the Advancement of Science.

**Figure 2.**
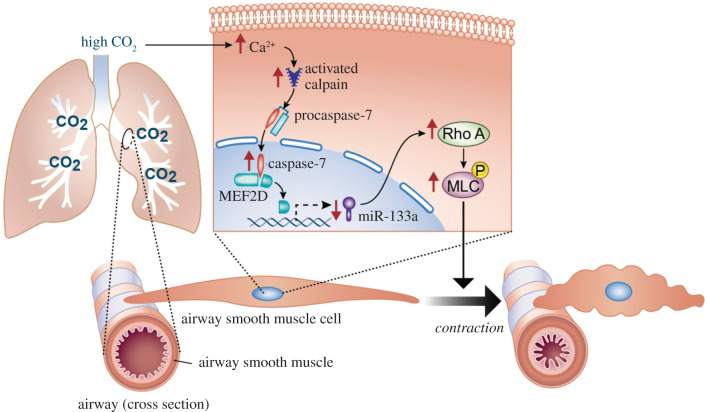
Schematic of calcium–calpain signalling in airway smooth muscle cell contraction during hypercapnia. Hypercapnia promotes airway smooth muscle contractility through an increase in intracellular calcium (Ca^2+^) and consequent activation of calpain which cleaves caspase-7. Cleaved caspase-7, in turn, cleaves the transcription factor myocyte enhancer factor 2D (MEF2D) that reduces miR-133a expression, thereby increasing Ras homologue family member A (RhoA) abundance and MLC phosphorylation. Reproduced from [19]. Copyright © 2018 American Association for the Advancement of Science.

Evidence suggesting that changes in the level of CO_2_ in the blood influence the airway tone was first reported by Einthoven in 1892 [40]. He described that inhalation of high concentrations of carbonic acid (CO_2_-rich mixtures) caused bronchoconstriction in dogs, which was confirmed in various models of normoxic hypercapnia-exposed dogs [41–45] and cats [46,47]. Airway tone is regulated by interaction of the sympathetic and parasympathetic nerves [46,62] and the stimulation of vagal efferent nerves can increase the tone, resulting in bronchoconstriction [46,62–64]. As the hypercapnia-induced bronchoconstriction was abolished by blocking the vagus nerve, it was understood to be dependent on the integrity of vagal conduction [40–44,46,47]. In healthy humans, there have been few reports describing that the inhalation of high CO_2_ concentrations decreases specific airway or pulmonary conductance which is the mathematical inverse of airway resistance [48,49]. The increases in airway resistance during high CO_2_ exposure were interpreted as extrathoracic airway narrowing [48] such as larynx narrowing [49], because the hypercapnic effect was not blocked by atropine or *β*_1_/*β*_2_ adrenergic receptor agonists. However, the direct studies of laryngeal resistance during high CO_2_ exposure indicated no change in anaesthetized animals [65] and healthy human subjects [66]. Furthermore, several reports of bronchoconstriction in the hypercapnia-exposed animals [42,44,46] showed that the blockage of the vagus nerve did not entirely abolish the bronchoconstrictor response to the high CO_2_ exposure. These reports suggest that other mechanisms can contribute to the airway response to hypercapnia. Recently, we have reported that CO_2_ operates as a signalling molecule that increases contraction of mouse and human airway smooth muscle cells [19]. We found that high concentrations of CO_2_, independently of hypoxia and extracellular pH, increased acetylcholine (ACh)-induced cell contraction, which is both time- and dose-dependent in cultured cells (figure 1*a*). In a murine model, the exposure to normoxic hypercapnia, particularly chronic hypercapnia, increased ACh-induced airway contraction in precision lung cut slices (figure 1*b*) as well as airway resistance (figure 1*c*). Furthermore, we found that, in a small cohort of patients with chronic COPD, patients with hypercapnia had higher airway resistance (figure 1*d*), which improved after correction of hypercapnia (figure 1*e*). Our study also provided novel insights into the molecular mechanisms by which hypercapnia promotes airway smooth muscle cell contractility via calcium–calpain signalling. The signalling was mediated by caspase-7, which by cleaving the transcription factor myocyte-specific enhancer factor 2D (MEF2D), leads to downregulation of the microRNA-133a (miR-133a) and consequent upregulation of Ras homologue family member (Rho) A and myosin light-chain (MLC) phosphorylation (figure 2). Our data suggest that the elevation of CO_2_ levels activates specific signal transduction pathways in airway smooth muscle cells, which results in deleterious changes in the airway tone, leading to bronchoconstriction. Taken together, these reports suggest that hypercapnia can contribute to airway constriction by activating vagus nerve and high CO_2_-responsive signal transduction pathways. In lung disease conditions, hypercapnia may worsen airway constriction and limit ventilation to poorly functioning lung units setting up a feedback loop that could culminate in respiratory failure.

## 3. Respiratory acidosis-related bronchodilation

There have been studies reporting that hypercapnia shows airway relaxation [50–61]. We have reported that airway smooth muscle relaxation occurs during acute hypercapnia-associated acidosis, but it was transient and modest [19]. We reason that hypercapnia may acutely contribute to bronchodilatation when the tone of airways is previously increased by various constrictor stimuli such as drugs [52–54], hypoventilation [55,67–69] or when the reduction of ventilation in one lung following the occlusion of its pulmonary artery leads to bronchoconstriction in response to local airway ischaemia [70] and hypocapnia [50–52,71]. The inhalation of high CO_2_ concentrations reduces constriction of airways as well as the tension developed by isolated bronchial rings caused by drugs such as 5-hydroxytryptamine [52–54]. It also reverses the airway constriction associated with pulmonary artery occlusion in ventilated animal models [50,52]. In humans, the administration of high CO_2_ can relax the constriction of airways in the patient with unilateral pulmonary artery occlusion [51] and young asthmatic adults with hyperventilation (hypocapnia) [55] or exercise-induced bronchoconstriction [55,56]. These *in vivo* and *in vitro* effects of hypercapnia were not stimulated by the nerve reflexes and were understood to be a result of changes in extracellular/intracellular pH level, possibly elevated CO_2_-related acidosis (respiratory acidosis) in airway smooth muscle cells. Many of the cellular responses to CO_2_ elevation are thought to be a consequence of acidosis because of the rapid conversion of CO_2_ in solution into H_2_CO_3_ and subsequently HCO_3_^−^ and H^+^ [5,72]. Several *in vitro* reports show that respiratory or normocapnic (metabolic) acidosis produced a reversible reduction in active tension of bronchial rings [53,54,57,58]. Extracellular pH can alter airway smooth muscle tone by changing the levels of pH and intracellular calcium (Ca^2+^) [58,59,73]. Intracellular acidification has been reported to decrease intracellular Ca^2+^ levels through voltage-dependent Ca^2+^ channels in the potassium-induced contractile model, thereby inhibiting airway smooth muscle cell contraction [60]. On the other hand, an *in vitro* study reported that high concentrations of CO_2_, independently of extracellular pH, enhanced airway smooth muscle relaxation via the epithelium-dependent mechanism induced by substance P in the model of methacholine-precontracted bronchial smooth muscle [61]. Collectively, elevated CO_2_ conditions, specifically showing acute respiratory acidosis, appear to have a potent relaxing effect on contracted airways.

## 4. Effect of hypocapnia on airway contractility

Low levels of CO_2_ (hypocapnia) have been also reported to increase airway constriction in humans with pulmonary artery occlusion [51,71], hyperventilation [67,68] and exercise-induced asthma attacks [55,56] and other models *in vivo* [50,52,70,74] and *in vitro* [59,61,75,76]. The bronchoconstrictor effect of hypocapnia is largely attributed to local mechanisms on the bronchial smooth muscle since it was not abolished by vagotomy or atropine in intact animals [50,70] and asthmatic patients [55,56]. Several reports suggest that the hypocapnic response involves additional contribution of cholinergic reflexes in the airways [67,68]. The cellular mechanisms involved in local airway response to hypocapnia are likely dependent on intracellular alkalosis elicited by hypocapnia on airway smooth muscle cells. *In vitro* studies suggest that intracellular alkalosis can increase airway smooth muscle contractility [77] by increasing intracellular Ca^2+^ levels through voltage-dependent calcium channels in airway smooth muscle [60,73,76].

## 5. Conclusion

**Figure 3.**
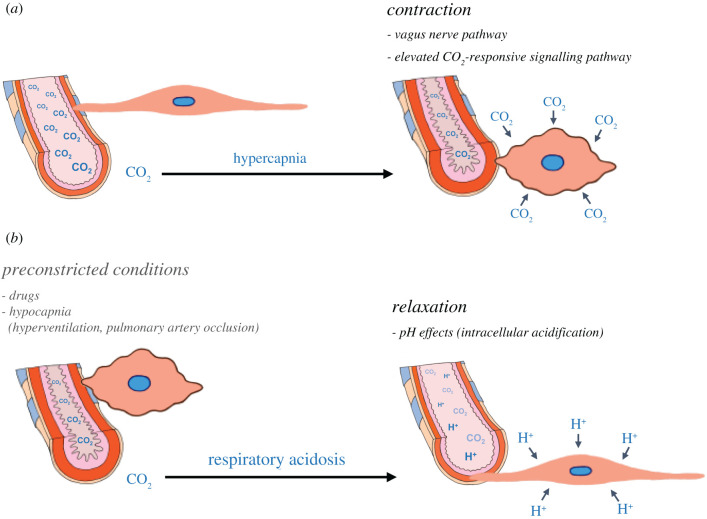
A proposed model for the modulation of CO_2_ in airway tone. Lung airway cells sense and respond to changes in CO_2_ levels, which modulates the tone of airways, airway contraction or relaxation, via specific mechanisms of the vagus reflexes, molecular CO_2_ and pH effects. (*a*) Hypercapnia. Acute and chronic hypercapnia promote airway contractility via either vagus reflexes or molecular CO_2_ effects. (*b*) Respiratory acidosis. Elevated CO_2_ conditions particularly showing acute respiratory acidosis can have a potent relaxing effect on contracted airways via pH effects.

A proposed model for the effects of CO_2_ levels on the airway tone, airway smooth muscle contractility or relaxation, is presented in figure 3. Lung airway cells appear to sense and respond to changes in CO_2_ levels via specific mechanisms of the vagus reflexes, molecular CO_2_ and pH effects. Thus, the effect of elevated CO_2_ levels to lung diseases is somewhat controversial. Hypercapnia is associated with worse outcomes in patients with obstructive lung diseases such as asthma [13], obesity hypoventilation syndrome [14] and COPD [10–12]. Furthermore, the recently reported strategy of mechanical ventilation aimed at reducing the partial pressure of CO_2_ in arterial blood can provide beneficial effects including improvement of airway resistance, health-related quality of life and mortality for patients with COPD and hypercapnia [11,12,19]. Understanding the elevated CO_2_ effects on airway contractility is of significant clinical interest for those patients and could help with the design of innovative therapeutic approaches.

## Data accessibility

This article has no additional data.

## References

[1] Weir EK, Lopez-Barneo J, Buckler KJ, Archer SL 2005 Acute oxygen-sensing mechanisms. N. Engl. J. Med. 353, 2042–2055. (10.1056/NEJMra050002) 16282179 PMC2803102

[2] Haldar SM, Stamler JS 2013 S-nitrosylation: integrator of cardiovascular performance and oxygen delivery. J. Clin. Invest. 123, 101–110. (10.1172/JCI62854) 23281416 PMC3533273

[3] Cummins EP, Strowitzki MJ, Taylor CT 2020 Mechanisms and consequences of oxygen and carbon dioxide sensing in mammals. Physiol. Rev. 100, 463–488. (10.1152/physrev.00003.2019) 31539306

[4] Cummins EP, Selfridge AC, Sporn PH, Sznajder JI, Taylor CT 2014 Carbon dioxide-sensing in organisms and its implications for human disease. Cell. Mol. Life Sci. 71, 831–845. (10.1007/s00018-013-1470-6) 24045706 PMC3945669

[5] Shigemura M, Lecuona E, Sznajder JI 2017 Effects of hypercapnia on the lung. J. Physiol. 595, 2431–2437. (10.1113/JP273781) 28044311 PMC5390878

[6] Monastersky R 2013 Global carbon dioxide levels near worrisome milestone. Nature 497, 13–14. (10.1038/497013a) 23636369

[7] Laffey JG, Kavanagh BP 1999 Carbon dioxide and the critically ill—too little of a good thing? Lancet 354, 1283–1286. (10.1016/S0140-6736(99)02388-0) 10520649

[8] ARDS Network. 2000 Ventilation with lower tidal volumes as compared with traditional tidal volumes for acute lung injury and the acute respiratory distress syndrome. The Acute Respiratory Distress Syndrome Network. N. Engl. J. Med. 342, 1301–1308. (10.1056/NEJM200005043421801) 10793162

[9] Nin N et al. 2017 Severe hypercapnia and outcome of mechanically ventilated patients with moderate or severe acute respiratory distress syndrome. Intensive Care Med. 43, 200–208. (10.1007/s00134-016-4611-1) 28108768 PMC5630225

[10] Connors AF et al. 1996 Outcomes following acute exacerbation of severe chronic obstructive lung disease. The SUPPORT investigators (Study to Understand Prognoses and Preferences for Outcomes and Risks of Treatments). Am. J. Respir. Crit. Care Med. 154, 959–967. (10.1164/ajrccm.154.4.8887592) 8887592

[11] Köhnlein T et al. 2014 Non-invasive positive pressure ventilation for the treatment of severe stable chronic obstructive pulmonary disease: a prospective, multicentre, randomised, controlled clinical trial. Lancet Respir. Med. 2, 698–705. (10.1016/S2213-2600(14)70153-5) 25066329

[12] Murphy PB et al. 2017 Effect of home noninvasive ventilation with oxygen therapy vs oxygen therapy alone on hospital readmission or death after an acute COPD exacerbation: a randomized clinical trial. JAMA 317, 2177–2186. (10.1001/jama.2017.4451) 28528348 PMC5710342

[13] Mountain RD, Sahn SA 1988 Clinical features and outcome in patients with acute asthma presenting with hypercapnia. Am. Rev. Respir. Dis. 138, 535–539. (10.1164/ajrccm/138.3.535) 3202409

[14] Piper A 2015 Obesity hypoventilation syndrome: weighing in on therapy options. Chest 149, 856–868. (10.1378/chest.15-0681) 26292036

[15] Bharat A, Graf N, Mullen A, Kanter J, Andrei AC, Sporn PH, DeCamp MM, Sznajder JI 2016 Pleural hypercarbia after lung surgery is associated with persistent alveolopleural fistulae. Chest 149, 220–227. (10.1378/chest.15-1591) 26402303 PMC5831571

[16] Shigemura M, Homma T, Sznajder JI 2020 Hypercapnia: an aggravating factor in asthma. J. Clin. Med. 9, 3207 (10.3390/jcm9103207) PMC7599850 33027886

[17] Vadasz I et al. 2008 AMP-activated protein kinase regulates CO_2_-induced alveolar epithelial dysfunction in rats and human cells by promoting Na,K-ATPase endocytosis. J. Clin. Invest. 118, 752–762. (10.1172/JCI29723) 18188452 PMC2176184

[18] Gates KL, Howell HA, Nair A, Vohwinkel CU, Welch LC, Beitel GJ, Hauser AR, Sznajder JI, Sporn PH 2013 Hypercapnia impairs lung neutrophil function and increases mortality in murine pseudomonas pneumonia. Am. J. Respir. Cell Mol. Biol. 49, 821–828. (10.1165/rcmb.2012-0487OC) 23777386 PMC3931098

[19] Shigemura M et al. 2018 Hypercapnia increases airway smooth muscle contractility via caspase-7-mediated miR-133a-RhoA signaling. Sci. Transl. Med. 10, eaat1662 (10.1126/scitranslmed.aat1662) 30185650 PMC6889079

[20] Jaitovich A et al. 2015 High CO_2_ levels cause skeletal muscle atrophy via AMP-activated kinase (AMPK), FoxO3a protein, and muscle-specific ring finger protein 1 (MuRF1). J. Biol. Chem. 290, 9183–9194. (10.1074/jbc.M114.625715) 25691571 PMC4423704

[21] Korponay TC, Balnis J, Vincent CE, Singer DV, Chopra A, Adam AP, Ginnan R, Singer HA, Jaitovich A 2020 High CO_2_ downregulates skeletal muscle protein anabolism via AMP-activated protein kinase alpha2-mediated depressed ribosomal biogenesis. Am. J. Respir. Cell Mol. Biol. 62, 74–86. (10.1165/rcmb.2019-0061OC) 31264907 PMC6938128

[22] Balnis J, Korponay TC, Jaitovich A 2020 AMP-activated protein kinase (AMPK) at the crossroads between CO_2_ retention and skeletal muscle dysfunction in chronic obstructive pulmonary disease (COPD). Int. J. Mol. Sci. 21, 955 (10.3390/ijms21030955) PMC7037951 32023946

[23] Wang N, Gates KL, Trejo H, Favoreto Jr S, Schleimer RP, Sznajder JI, Beitel GJ, Sporn PH 2010 Elevated CO_2_ selectively inhibits interleukin-6 and tumor necrosis factor expression and decreases phagocytosis in the macrophage. FASEB J. 24, 2178–2190. (10.1096/fj.09-136895) 20181940 PMC2887258

[24] Cummins EP et al. 2010 NF-kappaB links CO_2_ sensing to innate immunity and inflammation in mammalian cells. J. Immunol. 185, 4439–4445. (10.4049/jimmunol.1000701) 20817876

[25] Oliver KM, Lenihan CR, Bruning U, Cheong A, Laffey JG, McLoughlin P, Taylor CT, Cummins EP 2012 Hypercapnia induces cleavage and nuclear localization of RelB protein, giving insight into CO_2_ sensing and signaling. J. Biol. Chem. 287, 14 004–14 011. (10.1074/jbc.M112.347971) PMC3340129 22396550

[26] Casalino-Matsuda SM, Nair A, Beitel GJ, Gates KL, Sporn PH 2015 Hypercapnia inhibits autophagy and bacterial killing in human macrophages by increasing expression of Bcl-2 and Bcl-xL. J. Immunol. 194, 5388–5396. (10.4049/jimmunol.1500150) 25895534 PMC4433787

[27] Lu Z, Casalino-Matsuda SM, Nair A, Buchbinder A, Budinger GRS, Sporn PHS, Gates KL 2018 A role for heat shock factor 1 in hypercapnia-induced inhibition of inflammatory cytokine expression. FASEB J. 32, 3614–3622. (10.1096/fj.201701164R) 29405096 PMC5998969

[28] Casalino-Matsuda SM et al. 2018 Hypercapnia alters expression of immune response, nucleosome assembly and lipid metabolism genes in differentiated human bronchial epithelial cells. Sci. Rep. 8, 13508 (10.1038/s41598-018-32008-x) 30202079 PMC6131151

[29] Shigemura M et al. 2019 Elevated CO_2_ regulates the Wnt signaling pathway in mammals, *Drosophila melanogaster* and *Caenorhabditis elegans*. Sci. Rep. 9, 18251 (10.1038/s41598-019-54683-0) 31796806 PMC6890671

[30] Briva A et al. 2007 High CO_2_ levels impair alveolar epithelial function independently of pH. PLoS ONE 2, e1238 (10.1371/journal.pone.0001238) 18043745 PMC2077933

[31] Vadasz I et al. 2012 Evolutionary conserved role of c-Jun-N-terminal kinase in CO_2_-induced epithelial dysfunction. PLoS ONE 7, e46696 (10.1371/journal.pone.0046696) 23056407 PMC3466313

[32] Welch LC, Lecuona E, Briva A, Trejo HE, Dada LA, Sznajder JI 2010 Extracellular signal-regulated kinase (ERK) participates in the hypercapnia-induced Na,K-ATPase downregulation. FEBS Lett. 584, 3985–3989. (10.1016/j.febslet.2010.08.002) 20691686 PMC2966388

[33] Dada LA et al. 2015 High CO_2_ leads to Na,K-ATPase endocytosis via c-Jun amino-terminal kinase-induced LMO7b phosphorylation. Mol. Cell. Biol. 35, 3962–3973. (10.1128/MCB.00813-15) 26370512 PMC4628060

[34] Lecuona E, Sun H, Chen J, Trejo HE, Baker MA, Sznajder JI 2013 Protein kinase A-Ialpha regulates Na,K-ATPase endocytosis in alveolar epithelial cells exposed to high CO_2_ concentrations. Am. J. Respir. Cell Mol. Biol. 48, 626–634. (10.1165/rcmb.2012-0373OC) 23349050 PMC3707378

[35] Kryvenko V, Wessendorf M, Morty RE, Herold S, Seeger W, Vagin O, Dada LA, Sznajder JI, Vadasz I 2020 Hypercapnia impairs Na,K-ATPase function by inducing endoplasmic reticulum retention of the beta-subunit of the enzyme in alveolar epithelial cells. Int. J. Mol. Sci. 21, 1467 (10.3390/ijms21041467) PMC7073107 32098115

[36] Doerr CH, Gajic O, Berrios JC, Caples S, Abdel M, Lymp JF, Hubmayr RD 2005 Hypercapnic acidosis impairs plasma membrane wound resealing in ventilator-injured lungs. Am. J. Respir. Crit. Care Med. 171, 1371–1377. (10.1164/rccm.200309-1223OC) 15695495 PMC2718480

[37] O'Toole D, Hassett P, Contreras M, Higgins BD, McKeown ST, McAuley DF, O'Brien T, Laffey JG 2009 Hypercapnic acidosis attenuates pulmonary epithelial wound repair by an NF-kappaB dependent mechanism. Thorax 64, 976–982. (10.1136/thx.2008.110304) 19617214

[38] Vohwinkel CU, Lecuona E, Sun H, Sommer N, Vadász I, Chandel NS, Sznajder JI 2011 Elevated CO_2_ levels cause mitochondrial dysfunction and impair cell proliferation. J. Biol. Chem. 286, 37 067–37 076. (10.1074/jbc.M111.290056) PMC3199454 21903582

[39] Bharat A et al. 2020 High CO_2_ levels impair lung wound healing. Am. J. Respir. Cell Mol. Biol. 63, 244–254. (10.1165/rcmb.2019-0354OC) 32275835 PMC7397765

[40] Einthoven W 1892 Ueber die Wirkung der Bronchialmuskeln, nach einer neuen Methode untersucht, und über Asthma nervosum. Arch. Gesamte Physiol. Menschen Tiere 51, 367–445. (10.1007/BF01671026)

[41] De Burgh Daly M, Lambertsen DC, Schweitzer A 1953 The effects upon the bronchial musculature of altering the oxygen and carbon dioxide tensions of the blood perfusing the brain. J. Physiol. 119, 292–314. (10.1113/jphysiol.1953.sp004848) 13035754 PMC1392795

[42] Loofbourrow GN, Wood WB, Baird IL 1957 Tracheal constriction in the dog. Am. J. Physiol. 191, 411–415. (10.1152/ajplegacy.1957.191.2.411) 13478753

[43] Nadel JA, Widdicombe JG 1962 Effect of changes in blood gas tensions and carotid sinus pressure on tracheal volume and total lung resistance to airflow. J. Physiol. 163, 13–33. (10.1113/jphysiol.1962.sp006956) 14477795 PMC1359686

[44] Green M, Widdicombe JG 1966 The effects of ventilation of dogs with different gas mixtures on airway calibre and lung mechanics. J. Physiol. 186, 363–381. (10.1113/jphysiol.1966.sp008040) 5972114 PMC1395851

[45] Kondo T, Kobayashi I, Hayama N, Tazaki G, Ohta Y 2000 Role of cholinergic neural transmission on airway resistance in the dog. J. Auton. Nerv. Syst. 80, 64–70. (10.1016/s0165-1838(00)00079-5) 10742541

[46] Dixon WE 1903 Contributions to the physiology of the lungs: Part I. The bronchial muscles, their innervation, and the action of drugs upon them. J. Physiol. 29, 97–173. (10.1113/jphysiol.1903.sp000947) 16992663 PMC1540618

[47] Iscoe S, Fisher JT 1995 Bronchomotor responses to hypoxia and hypercapnia in decerebrate cats. J. Appl. Physiol. (1985) 78, 117–123. (10.1152/jappl.1995.78.1.117) 7713800

[48] Sterling GM 1969 The mechanism of decreased specific airway conductance in man during hypercapnia caused by inhalation of 7 per cent CO_2_. Clin. Sci. 37, 539–548. 5359006

[49] Rodarte JR, Hyatt RE 1973 Effect of acute exposure to CO_2_ on lung mechanics in normal man. Respir. Physiol. 17, 135–145. (10.1016/0034-5687(73)90057-1) 4689451

[50] Severinghaus JW, Swenson EW, Finley TN, Lategola MT, Williams J 1961 Unilateral hypoventilation produced in dogs by occluding one pulmonary artery. J. Appl. Physiol. 16, 53–60. (10.1152/jappl.1961.16.1.53) 13750428

[51] Darke CS, Astin TW 1972 Differential ventilation in unilateral pulmonary artery occlusion. Thorax 27, 480–486. (10.1136/thx.27.4.480) 5075620 PMC469955

[52] Astin TW, Barer GR, Shaw JW, Warren PM 1973 The action of carbon dioxide on constricted airways. J. Physiol. 235, 607–623. (10.1113/jphysiol.1973.sp010407) 4772402 PMC1350783

[53] Sterling GM, Holst PE, Nadel JA 1972 Effect of CO_2_ and pH on bronchoconstriction caused by serotonin vs. acetylcholine. J. Appl. Physiol. 32, 39–43. (10.1152/jappl.1972.32.1.39) 5007015

[54] Duckles SP, Rayner MD, Nadel JA 1974 Effects of CO_2_ and pH on drug-induced contractions of airway smooth muscle. J. Pharmacol. Exp. Ther. 190, 472–481. 4413181

[55] Fisher HK, Holton P, Buxton RS, Nadel JA 1970 Resistance to breathing during exercise-induced asthma attacks. Am. Rev. Respir. Dis. 101, 885–896. (10.1164/arrd.1970.101.6.885) 5419973

[56] Fisher HK, Hansen TA 1976 Site of action of inhaled 6 per cent carbon dioxide in the lungs of asthmatic subjects before and after exercise. Am. Rev. Respir. Dis. 114, 861–870. (10.1164/arrd.1976.114.5.861) 984580

[57] Stephens NL, Meyers JL, Cherniack RM 1968 Oxygen, carbon dioxide, H^+^ ion, and bronchial length-tension relationships. J. Appl. Physiol. 25, 376–383. (10.1152/jappl.1968.25.4.376)

[58] Croxton TL, Lande B, Hirshman CA 1995 Role of intracellular pH in relaxation of porcine tracheal smooth muscle by respiratory gases. Am. J. Physiol. 268, L207–L213. (10.1152/ajplung.1995.268.2.L207) 7864141

[59] Twort CH, Cameron IR 1986 Effects of PCO_2_, pH and extracellular calcium on contraction of airway smooth muscle from rats. Respir. Physiol. 66, 259–267. (10.1016/0034-5687(86)90078-2) 3099356

[60] Yamakage M, Lindeman KS, Hirshman CA, Croxton TL 1995 Intracellular pH regulates voltage-dependent Ca^2+^ channels in porcine tracheal smooth muscle cells. Am. J. Physiol. 268, L642–L646. (10.1152/ajplung.1995.268.4.l642) 7733305

[61] El Mays TY, Saifeddine M, Choudhury P, Hollenberg MD, Green FH 2011 Carbon dioxide enhances substance P-induced epithelium-dependent bronchial smooth muscle relaxation in Sprague-Dawley rats. Can. J. Physiol. Pharmacol. 89, 513–520. (10.1139/Y11-052) 21812529

[62] Cabezas GA, Graf PD, Nadel JA 1971 Sympathetic versus parasympathetic nervous regulation of airways in dogs. J. Appl. Physiol. 31, 651–655. (10.1152/jappl.1971.31.5.651) 4399001

[63] Colebatch HJ, Halmagyi DF 1963 Effect of vagotomy and vagal stimulation on lung mechanics and circulation. J. Appl. Physiol. 18, 881–887. (10.1152/jappl.1963.18.5.881) 14063255

[64] Woolcock AJ, Macklem PT, Hogg JC, Wilson NJ, Nadel JA, Frank NR, Brain J 1969 Effect of vagal stimulation on central and peripheral airways in dogs. J. Appl. Physiol. 26, 806–813. (10.1152/jappl.1969.26.6.806) 5786412

[65] Campbell CJ, Murtagh JA, Raber CF 1963 Chemical agents and reflex control of laryngeal glottis. Ann. Otol. Rhinol. Laryngol. 72, 589–604. (10.1177/000348946307200301) 14062414

[66] Spann RW, Hyatt RE 1971 Factors affecting upper airway resistance in conscious man. J. Appl. Physiol. 31, 708–712. (10.1152/jappl.1971.31.5.708) 5117185

[67] Newhouse MT, Becklake MR, Macklem PT, McGregor M 1964 Effect of alterations in end-tidal CO_2_ tension on flow resistance. J. Appl. Physiol. 19, 745–749. (10.1152/jappl.1964.19.4.745) 14195587

[68] Sterling GM 1968 The mechanism of bronchoconstriction due to hypocapnia in man. Clin. Sci. 34, 277–285. 5653688

[69] Nielsen TM, Pedersen OF 1976 The effect of CO_2_ on peripheral airways. Acta Physiol. Scand. 98, 192–199. 983728

[70] Tisi GM, Wolfe WG, Fallat RJ, Nadel JA 1970 Effects of O_2_ and CO_2_ on airway smooth muscle following pulmonary vascular occlusion. J. Appl. Physiol. 28, 570–573. (10.1152/jappl.1970.28.5.570) 4315146

[71] Swenson EW, Finley TN, Guzman SV 1961 Unilateral hypoventilation in man during temporary occlusion of one pulmonary artery. J. Clin. Invest. 40, 828–835. (10.1172/JCI104316) 13774279 PMC290794

[72] Casey JR, Grinstein S, Orlowski J 2010 Sensors and regulators of intracellular pH. Nat. Rev. Mol. Cell Biol. 11, 50–61. (10.1038/nrm2820) 19997129

[73] Yamakage M, Kohro S, Yamauchi M, Namiki A 1995 The effects of extracellular pH on intracellular pH, Ca^2+^ and tension of canine tracheal smooth muscle strips. Life Sci. 56, PL175–PL180. (10.1016/0024-3205(94)00497-g) 7869833

[74] Kikuchi R, Kikuchi K, Hildebrandt J, Yanai M, Sekizawa K, Sasaki H 1995 Dependence of collateral and small airway resistances of CO_2_ and volume in dog lobes. Respir. Physiol. 100, 245–252. (10.1016/0034-5687(94)00137-o) 7481114

[75] Duane SF, Weir EK, Stewart RM, Niewoehner DE 1979 Distal airway responses to changes in oxygen and carbon dioxide tensions. Respir. Physiol. 38, 303–311. (10.1016/0034-5687(79)90056-2) 523847

[76] Lindeman KS, Croxton TL, Lande B, Hirshman CA 1998 Hypocapnia-induced contraction of porcine airway smooth muscle. Eur. Respir. J. 12, 1046–1052. (10.1183/09031936.98.12051046) 9863995

[77] Stephens NL, Mitchell RW 1977 Effect of respiratory acidosis and activity on airway smooth muscle intracellular pH. J. Appl. Physiol. Respir. Environ. Exerc. Physiol. 42, 408–412. (10.1152/jappl.1977.42.3.408) 14103

